# Early preterm infant microbiome impacts adult learning

**DOI:** 10.1038/s41598-022-07245-w

**Published:** 2022-02-28

**Authors:** Jing Lu, Lei Lu, Yueyue Yu, Kaitlyn Oliphant, Alexander Drobyshevsky, Erika C. Claud

**Affiliations:** 1 Department of Pediatrics, Pritzker School of Medicine/Biological Sciences Division, University of Chicago, Chicago, IL 60637 USA; 2 Department of Pediatrics, NorthShore University HealthSystem Research Institute, Evanston, IL 60202 USA

**Keywords:** Microbiology, Neuroscience

## Abstract

Interventions to mitigate long-term neurodevelopmental deficits such as memory and learning impairment in preterm infants are warranted. Manipulation of the gut microbiome affects host behaviors. In this study we determined whether early maturation of the infant microbiome is associated with neurodevelopment outcomes. Germ free mice colonized at birth with human preterm infant microbiomes from infants of advancing post menstrual age (PMA) demonstrated an increase in bacterial diversity and a shift in dominance of taxa mimicking the human preterm microbiome development pattern. These characteristics along with changes in a number of metabolites as the microbiome matured influenced associative learning and memory but not locomotor ability, anxiety-like behaviors, or social interaction in adult mice. As a regulator of learning and memory, brain glial cell-derived neurotrophic factor increased with advancing PMA and was also associated with better performance in associative learning and memory in adult mice. We conclude that maturation of the microbiome in early life of preterm infants primes adult associative memory and learning ability. Our findings suggest a critical window of early intervention to affect maturation of the preterm infant microbiome and ultimately improve neurodevelopmental outcomes.

## Introduction

The incidence of neurodevelopmental disabilities in preterm infants is rising despite the increasing survival rate of preterm infants and the collective efforts to improve both maternal and neonatal care^1–3^. Follow-up studies have shown that when evaluated at 3–4 years of age and compared to those who were born at term, prematurely born children (gestational age (GA) between 29 and 34 weeks) had specific deficits in sustained attention, visuospatial processing, and spatial working memory^4^. As revealed over the years, one of the leading factors affecting the pathogenesis of an array of preterm infant morbidities, namely necrotizing enterocolitis (NEC) and sepsis, is the immature microbiome pattern colonized in the gut^5^. Acute systemic inflammation caused by infection, prevalent in preterm infants with NEC and sepsis, can lead to one of the most distinctive cognitive deficits: memory and learning impairment^6^. Such memory loss manifests as impaired explicit recall in humans and deficiencies of fear-associated memory and reduced performance for object-recognition tasks in laboratory animals^6,7^. These studies and others therefore suggest that the potential effects of gut microbiota on brain functions could be targeted to improve the neurodevelopmental outcomes of the preterm infants^8–11^.

After initial colonization, a newborn’s microbiome develops over the course of the first 2–3 years of life, which parallels the maturing process of the neonatal brain^12,13^. A recent human study has demonstrated that infants born > 2500 g and later than 37 weeks of GA with increased richness and reduced evenness, lower abundance of *Bacteroides*, increased abundance of *Veillonella*, *Dialister*, and *Clostridiales* of the 1-month microbiome display increased non-social fear^14^. The impacts of gut microbiota of preterm infants, who have known increased risk for neurological disorders, on brain development and functions, however, has not been extensively studied. One intriguing observation emerging from animal studies suggests that manipulation of gut microbiota in the early stages of brain development is most likely to have an effect on behaviors later in life^15–18^. Current studies have revealed that different from term infants, GA and postmenstrual age (PMA) are the dominant factors in preterm microbiome assembly; independent of confounders such as mode of delivery, breastfeeding duration and antibiotic exposure^19^, which all play important roles in determining the colonization and assembly of the microbiome in term infants. We therefore hypothesize that initial colonization and microbiota development of the preterm infant microbiome, has distinct effects on the risk of developing deficits later in life.

In this study, we transfaunated fecal samples from human preterm infants born at 27–34 weeks GA to pregnant C57/BL6J germ free (GF) dams in a gnotobiotic environment. This allowed us to investigate brain development under the “isolated” influence of the preterm microbiome at the earliest stages of colonization and assembly. Offspring neurodevelopment was evaluated by behavioral testing at 12 weeks of age (adult) after colonization with the respective microbiome representing different PMA. PMA was defined in this study as GA+ weeks of life of donor samples collected and actually used to colonize the mice. The composition of the respective microbiome and the metabolites in the fecal and serum were analyzed to identify the significant microbial markers influencing brain functions.

## Results

### Diversity and taxonomic analysis of microbiota

**Figure 1:**
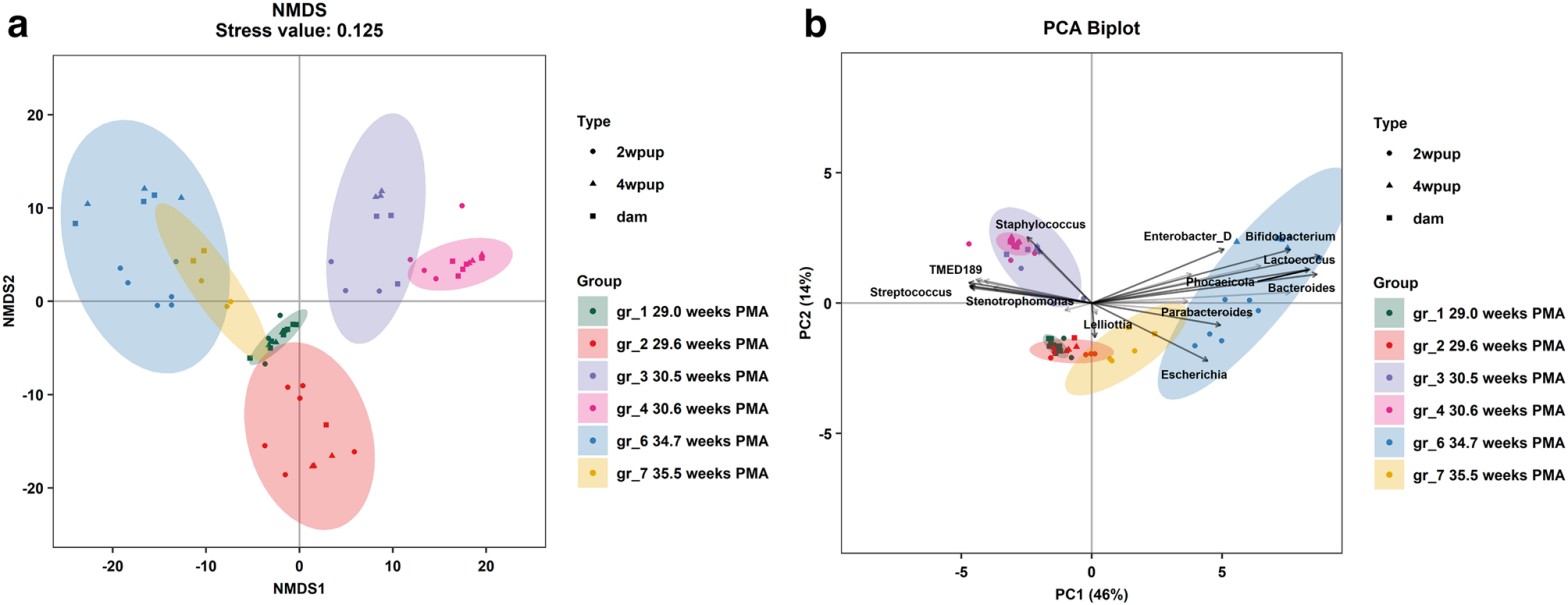
Microbial analysis of fecal samples from dam, 2 weeks old (2wpup) and 4 weeks old pups (4wpup). (**a**) Significant separation in the gut microbiome composition (beta-diversity) was observed among different transfaunation groups (gr) by PERMANOVA (color, *p* = 0.001) and no differences were detected among sample types (shape: 2wpup, 4wpup and dam) while controlling for group (PERMANOVA, *p* = 0.071). NMDS plot was generated using Aitchison distance metric at genus level. (**b**) Principal Component Analysis (PCA) biplot showed the variation among the fecal samples based on type and transfaunation groups and the relative abundance of microbial taxa (genera). Arrows represent the strength (through the length) of each taxa to the overall distribution.

To first confirm the fidelity of the colonization model, fecal samples were collected from dams colonized with human fecal samples from preterm infants with varying PMA (see Table 1 for clinical characteristics) at E20-21 (within a day of delivery), and from pups at 2 and 4 weeks of age for 16S rRNA sequencing analysis. NMDS analysis of fecal samples revealed that there was no distinction in fecal samples based on the time of sampling (indicated by different shapes in Fig. 1a), but there was a differentiation in fecal samples based on the transfaunation group (gr) of the sample (indicated by different colors in Fig. 1a). Gr_1-7 corresponded to animals colonized with fecal samples of patient 1–7 in Table 1. Gr_5 was excluded in the analysis due to missing samples at 2 and 4 weeks of age.

**Table 1:** Clinical characteristics of the donors of fecal samples.

**Patient characteristics**							
Patient number	1	2	3	4	5	6	7
Gestational age, weeks	27.0	28.2	28.4	29.6	32.4	33.9	34.0
Postmenstrual age, weeks	29.0	29.6	30.5	30.6	34.4	34.7	35.5
Day of life, days	14	10	14	7	14	6	11
Birth weight, kg	1.1	1.1	1.3	1.1	1.6	2	1.9
**Patient outcomes**							
Days of antibiotics after first 48 h of life	0	12	2	5	1	0	0.5
Days of breastmilk in the first 2 weeks of life	14	N/A	14	14	14	12	14
Days of total parenteral nutrition	15	20	35	5	7	N/A	4
Gestational age at discharge, weeks	38	39	40	40	36	36	36
Neonatal necrotizing enterocolitis (NEC)	No	No	No	No	No	No	No
Bronchopulmonary dysplasia (BPD)	Yes	No	Yes	No	No	No	No
Positive blood culture	No	No	No	No	No	No	No
Grade III/IV intraventricular hemorrhage (IVH)	No	No	Yes	No	No	No	No
Periventricular hemorrhage (PVL)	No	No	No	No	No	No	No
Seizures	No	No	No	No	No	No	No

PERMANOVA analysis showed that the dam and her respective 2 and 4 weeks old pups clustered together with no significant difference in beta-diversity within sampling time (dam, 2 weeks pups, 4 weeks pups) when controlling for transfaunation group (*p* = 0.071). However, PERMANOVA p-value for difference in beta-diversity by transfaunation group was statistically significant for the 2 weeks old pups (*p* = 0.001), 4 weeks old pups (*p* = 0.001) and dam (*p* = 0.001) samples, respectively, demonstrating that there were compositional differences among the transfaunation groups. In particular, PCA loading plot (Fig. 1b) demonstrated that transfaunation Groups_1, 2, 3, and 4 separate towards the leftmost direction and this separation was driven by the presence of *Staphylococcus*, *Streptococcus*, and *Stenotrophomonas*. Notably, groups_1,2.3, and 4 were from infants born less than 30 weeks of GA. Transfaunation Groups 6 and 7 (from infants born greater than 30 weeks of GA) separated in the rightmost direction from the other samples, and the features that largely contributed to the separation in that direction were *Enterobacter_D, Bifidobacterium, Lactococcus, Phocaeicola, Parabacteroides,* and *Escherichia*. These data largely agreed with the previously demonstrated succession order of microbiome colonization in preterm infant with class Bacilli (mostly genera *Staphylococcus* and *Streptococcus* in our study) dominating between 25–30 weeks of GA, followed by γ-Proteobacteria (genus *Enterobacter*_*D* in our study) and later Actinobacteria (genus *Bifidobacterium* in our study) between GA of 30–35 weeks^5,20^.

We observed significant increases in number of observed species (Richness) as the PMA increased in fecal samples of dams (Supplementary Fig. S1a, *p* = 0.02), 2 weeks (Supplementary Fig. S1b, *p* = 0.03) and but not 4 weeks old pups (Supplementary Fig. S1c, *p* = 0.06). Shannon diversity was positively correlated with PMA (Supplementary Fig. S1d, *p* = 0.03) in the fecal samples from the dams, but not 2 weeks old pups (Supplementary Fig. S1e, *p* = 0.09) and 4 weeks old samples (Supplementary Fig. S1f, *p* = 0.06). These data demonstrate that increased PMA of donors predicted a gradual increase of alpha-diversity (richness) in colonized dam and pups accordingly. Together with the beta-diversity results, our observation suggests a PMA-dependent maturation of microbiota reflected in the respective groups of transfaunated mice.

**Figure 2:**
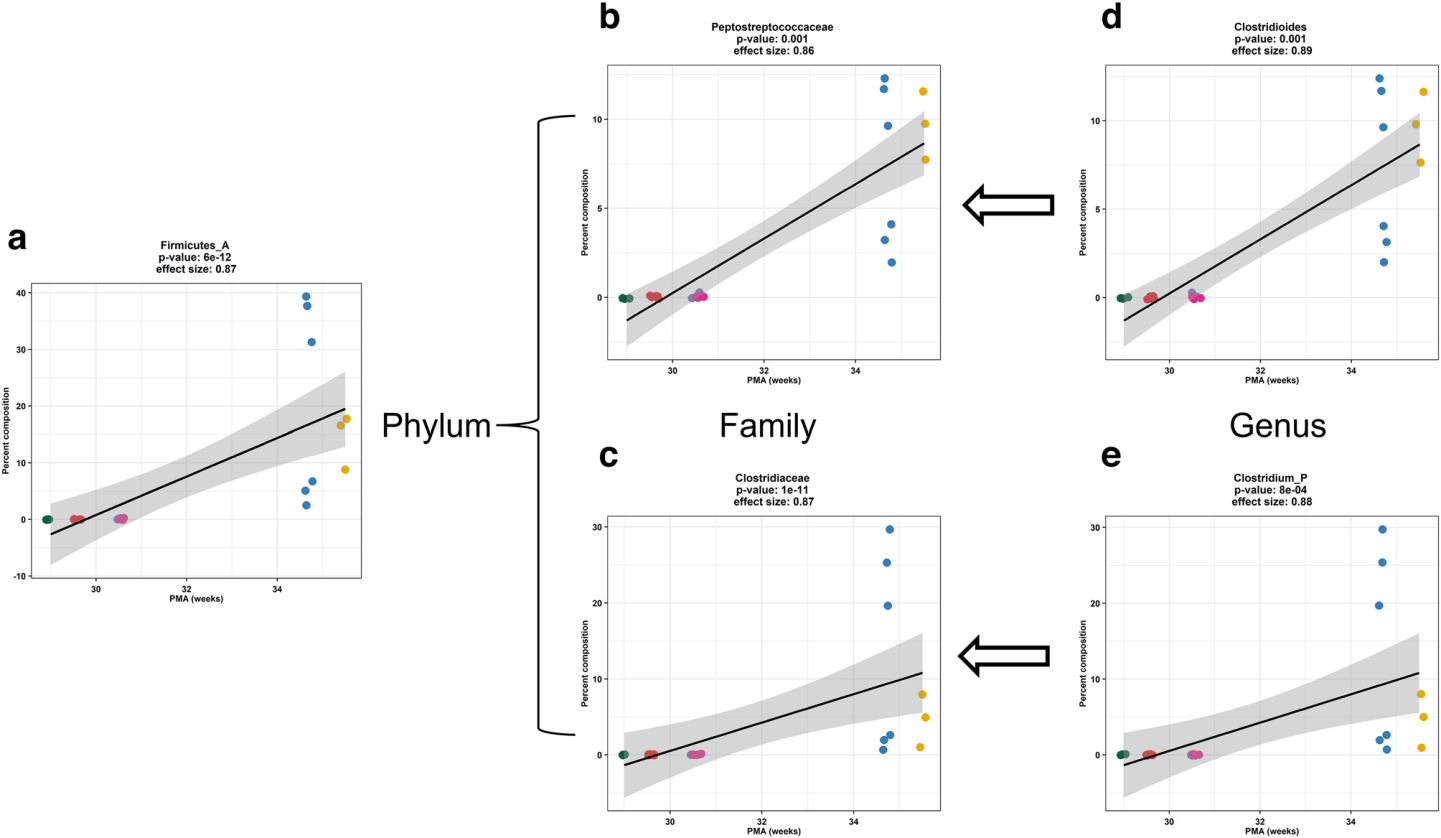
Bacterial families and genus contributed to PMA-dependent *Firmicutes_A* abundance in 2 weeks old fecal samples. At phylum level, PMA drove the *Firmicutes_A* relative abundance in 2 weeks old fecal samples. The relative abundance of *Firmicutes_A* based on operational taxonomic units (OTU) was significantly correlated with PMA (**a**, Pearson correlation *p* < 0.001). At family level, increased PMA positively correlated with the relative abundance (OTUs) of *Peptostreptococcaceae* and *Clostridiaceae* (**b**,**c**, Pearson correlation *p* < 0.01 and *p* < 0.01, respectively) from phylum *Firmicutes_A* in the 2 weeks old pup fecal samples. At genus level, the significant family difference in *Peptostreptococcaceae* abundance was related to *Clostridioides* and increased with PMA (**d**, Pearson correlation *p* < 0.001). The significant difference in *Clostridiaceae* abundance was related to *Clostridium_P* and the increase of *Clostridium_P* abundance was associated with and PMA (**e**, Pearson correlation *p* < 0.001).

**Figure 3:**
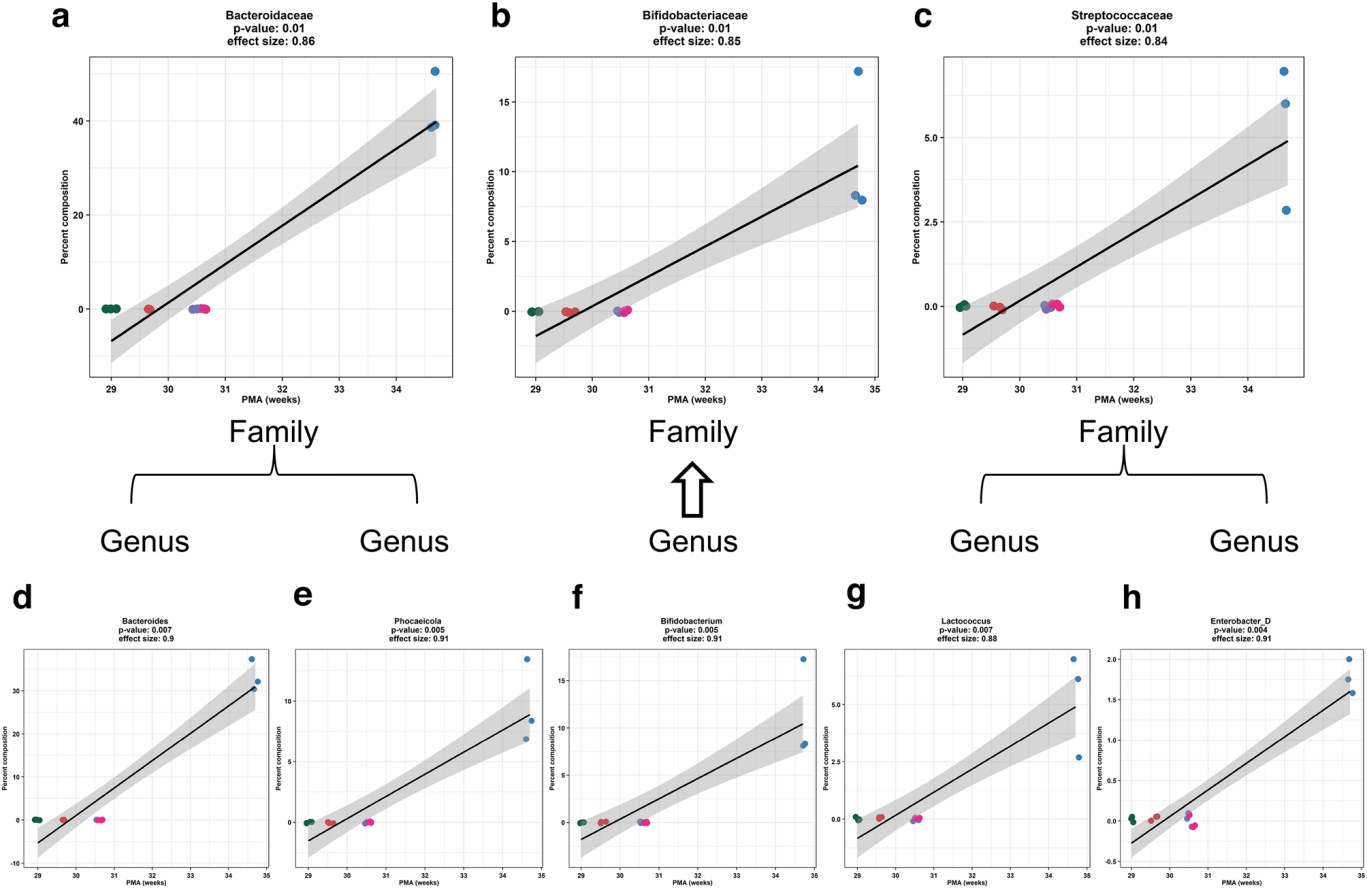
Distinct bacterial family and genus abundance in 4 weeks old fecal samples. For the 4 weeks old samples, the relative abundance (OTUs) of three families (*Bacteroidaceae*, *Bifidobacteriaceae*, and *Streptococcaceae*) increased with PMA (**a**–**c**). Five genera (*Bacteroides, Phocaeicola, Bifidobacterium, Lactococcus, Enterobacter_D*) were positively enhanced with PMA (**d**–**i**). All reached significance at *p* < 0.05 by Pearson correlation analysis.

We further observed a significant correlation between the relative abundance of the phylum *Firmicutes_A* and PMA (Fig. 2a) at 2 weeks of age based on operational taxonomic unit analysis. Two bacterial families *Peptostreptococcaceae* and *Clostridiaceae* contributed to the significant difference in *Firmicutes_A* abundance and significantly changed with PMA (Fig. 2b,c, respectively) in the 2 weeks old pup fecal samples. At the genus level, the significant family difference in *Peptostreptococcaceae* abundance was related to *Clostridioides* and increased with later PMA (Fig. 2d). The significant difference in *Clostridiaceae* abundance was related to *Clostridium_P* and the increase of *Clostridium_P* abundance was associated with later PMA (Fig. 2e). For the 4 weeks old samples, the abundance of three families (*Bacteroidaceae*, *Bifidobacteriaceae*, and *Streptococcaceae*) (Fig. 3a–c) and five genera (*Bacteroides, Phocaeicola, Bifidobacterium, Lactococcus, Enterobacter_D*) changed with PMA (Fig. 3d–h). These data further demonstrated that microbiota of 4 week old pups reflected the PMA-dependent early microbiome development highlighted by the increased abundance of the *Bacteroidaceae*, *Bifidobacteriaceae*, and *Streptococcaceae* bacterial families*.*

### Maturation of early microbiota was associated with better learning and memory but did not affect locomotor activity, anxiety-like behaviors and social interaction

Given that we have previously shown that microbiota affects behaviors^10^, we conducted a series of behavioral testing to evaluate locomotor development, anxiety-like behaviors, social behaviors and associative fear learning and memory. We first tested whether the early microbiota from preterm infants with different PMA could potentially affect adult locomotor activity and anxiety-like behaviors using the open field and elevated-plus maze tests. We did not observe any correlation between the average speed, time in the border area and the center area and PMA (Supplementary Fig. S2a–c) at 12 weeks of age. We also did not detect any correlation between the time spent in closed arm or the time spent in the open arm and PMA in the elevated-plus maze test (Supplementary Fig. S2d and S2e, respectively).

We further investigated whether the maturation of microbiota signified by PMA affects social interactions in the three-chamber social test. In the sociability test for social versus empty cage preference, there was no significant correlation between PMA and the ratio of the time pups spent in the chamber with a stranger vs an empty cage (Supplementary Fig. S3a). In the test for social novelty, PMA also did not affect the ratio of the time pups spent in the chamber with a strange vs a familiar partner (Supplementary Fig. S3b).

**Figure 4:**
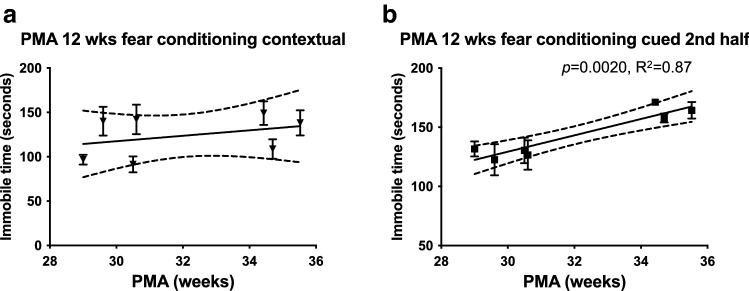
Increased PMA were positively correlated with fear learning and memory in the fear conditioning test. In the contextual memory test, the immobile time was not associated with increased PMA (**a**). In the cued fear retention test, the immobile time was significantly increased with increased PMA (**b**) in the second part of the cued fear conditioning test (different chamber and sound cue presented) by Pearson's correlation coefficient analysis. The coefficient of determination (R^2^) is also presented when significant correlation was reached at *p* < 0.05. Animal numbers used in each transfaunation groups were: gr_1 = 36, gr_2 = 8, gr_3 = 14, gr_4 = 6, gr_5 = 9, gr_6 = 11, and gr_7 = 7.

Fear conditioning test was then conducted to assess associative fear learning and memory. During the contextual test trial, we observed that immobile time in the same environment was not correlated with PMA at 12 weeks of age (Fig. 4a). However, in the second half of the cued test trial when the animals were placed in the new environment and the conditioned stimulus (CS: the auditory cue) was given without the unconditioned stimulus (US: the electric foot shock), PMA was significantly positively correlated with the immobile time at 12 weeks of age (Fig. 4b, p < 0.01, Pearson’s *r*^2^ = 0.87), indicating a difference in fear learning and memory.

### Discrete characteristics of the microbiota and cognitive function

**Figure 5:**
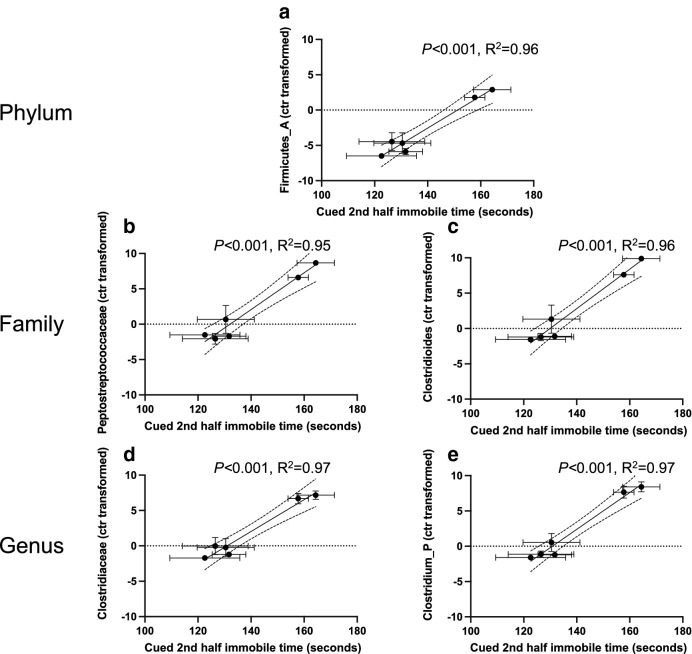
Specific bacterial taxonomic composition at 2 weeks old promoted cued fear retention. The relative abundance of *Firmicute_A* (**a**), *Peptostreptococcaceae* (**b**) and *Clostridiaceae* (**c**), *Clostridioides* (**d**) and *Clostridium_P* (**e**) in the fecal samples of 2 weeks old mice was significantly correlated with the immobile time in the second half of the cued fear conditioning test. Pearson's correlation coefficient was considered significant at *p* < 0.05.

**Figure 6:**
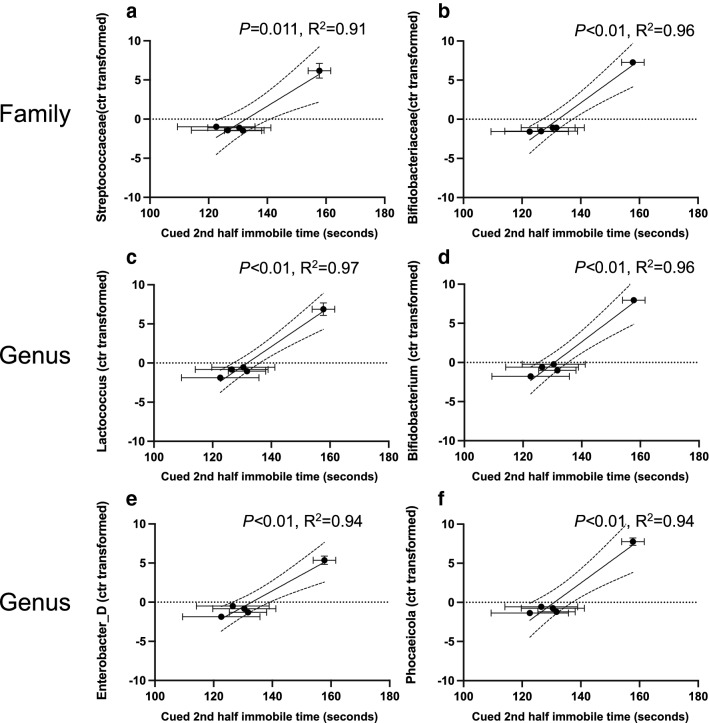
Specific bacterial taxonomic composition at 4 weeks old promoted cued fear retention. The relative abundance of *Streptococcaceae* (**a**) and *Bifidobacteriaceae* (**b**), *Lactococcus* (**c**)*, Bifidobacterium* (**d**)*, Enterobacter_D* (**e**) and *Phocaeicola* (**f**) in the fecal samples of 4 weeks old mice and the immobile behavior were positively correlated (all reached *p* < 0.05 by Pearson's correlation analysis).

We then attempted to identify the discrete characteristics of the PMA-dependent microbiota maturation that affect the above observed fear learning and memory. The increasing immobile time in the 2nd half of the cued test was significantly correlated with the increased richness of fecal samples from 2 (Supplementary Fig. S4a) and 4 (Supplementary Fig. S4b) weeks old pups. The immobile time also increased with the Shannon diversity of fecal samples from 4 weeks old pups (Supplementary Fig. S4d), but not from 2 weeks old pups (Supplementary Fig. S4c). Furthermore, at 2 weeks of age, the higher relative abundances of phylum *Firmicute_A* (Fig. 5a), family *Peptostreptococcaceae* (Fig. 5b) and *Clostridiaceae* (Fig. 5c), genus *Clostridioides* (Fig. 5d) and *Clostridium_P* (Fig. 5e) were correlated with higher immobile time in the fear conditioning test. Two PMA-depended families *Streptococcaceae* (Fig. 6a) and *Bifidobacteriaceae* (Fig. 6b), and four genera *Lactococcus* (Fig. 6c)*, Bifidobacterium* (Fig. 6d)*, Enterobacter_D* (Fig. 6e) and *Phocaeicola* (Fig. 6f) at 4 weeks of age were also positively correlated with the immobile behavior. These data demonstrate that the maturation of early microbiome signified by PMA, alpha diversity, and taxonomic features improved associative fear learning and memory in adult mice.

### Alterations in fecal and serum microbial metabolites were associated with microbial maturation-dependent learning and memory

To further identify the underlying mechanisms by which microbiota regulate the cognitive function we observed in the fear conditioning test, we analyzed fecal and serum metabolites from pups at 4 weeks of age colonized with fecal samples from four different PMAs (see Supplementary Table S1). Group_4 and 2 with corresponding 29.6 and 30.6 weeks PMA (see Table 1) were defined as the early group and Group_6 and 7 in Table 1 corresponding to 34.7 and 35.5 weeks PMA were defined as the late group. The mouse fecal dataset comprised a total of 1004 biochemicals, 813 named biochemicals and 191 unnamed biochemicals. The mouse serum dataset comprises a total of 847 biochemicals, 753 named biochemicals and 94 unnamed biochemicals.

**Figure 7:**
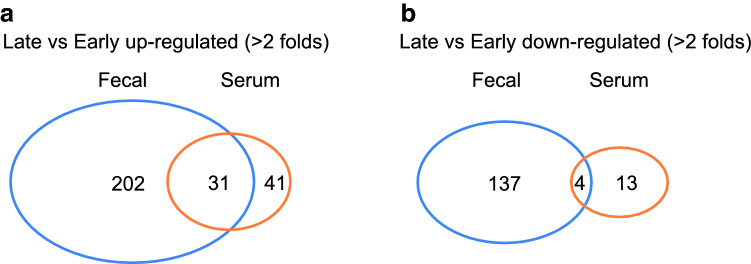
Overview of metabolomics. Venn diagram depicted the number of upregulated (**a**, > 2 folds), down-regulated (**b**, > 2 folds), and overlapping metabolites in fecal and serum samples between late PMA (> 30 weeks) and early PMA (< 30 weeks). Blue circles, metabolites from fecal samples. Red circles, metabolites from serum samples.

In total, there were 202 and 41 upregulated fecal and serum metabolites, respectively (late vs early > twofold changed based on ScaIedIMP value) (see Supplemental data Table S2 and Table S3) (Fig. 7a). There were also 137 and 13 downregulated (> twofold) fecal and serum metabolites, respectively (Fig. 7b). 31 of the upregulated and four of the downregulated metabolites were present in both fecal and serum sample pools.

First, Pearson correlation analysis (Supplementary Table S4) demonstrated that out of the 31 upregulated metabolites, the relative abundance levels (measured by area under the curve and presented as ScaledIMP ± SEM) of fecal 5-aminovalerate, phenol sulfate, 2,3-dihydroxy-2-methylbutyrate, ferulate, and ursodeoxycholate and the PMA (weeks) were positively correlated. In addition, PMA (weeks) was also positively correlated with serum nicotinate ribonucleoside and beta-muricholate. Both fecal and serum glutarate (C5-DC), cholate, phenylacetylglycine, ferulic acid 4 sulfate, and 2-oxindole-3-acetate were positively correlated with PMA.

Of the 31 upregulated common metabolites, 13 metabolites were associated with improved learning and memory (Table 2). The super biochemical pathways linked to the change of the fear conditioning behavior appeared to be amino acid metabolism, lipid metabolism and xenobiotics metabolism in both pools while cofactor and vitamin metabolism in the serum pool were also altered (Table 2). The relative abundance (ScaledIMP ± SEM) of five fecal metabolites (Supplementary Fig. S5a, 5-aminovalerate; S5b, phenol sulfate; S5c, 2,3-dihydroxy-2-methylbutyrate; S5d, ferulate; S5e, ursodeoxycholate) and six serum metabolites (Supplementary Fig. S6a, nicotinate ribonucleoside; S6b, cholate; S6c, beta-muricholate; S6d, ferulic acid 4 sulfate; S6e, 2-oxindole-3-acetate; and S6f, 4-methylcatechol sulfate), two from both pools (Supplementary Fig. S7a and S7b, fecal and serum phenylacetylglycine, respectively and Fig. S7c and S7d, fecal and serum glutarate (C5-DC), respectively) were associated with the immobile time in the 2nd half cued test in the fear conditioning test. None of the four downregulated metabolites present in both pools were associated with the behavioral results.

**Table 2:** Fecal and serum metabolites and pathways associated with the alteration of behavior.

	Biochemical	Super pathway	Sub pathway
Fecal only	5-Aminovalerate	Amino acid	Lysine metabolism
	Phenol sulfate	Amino acid	Tyrosine metabolism
	2,3-Dihydroxy-2-methylbutyrate	Amino acid	Leucine, isoleucine and valine metabolism
	Ursodeoxycholate	Lipid	Secondary bile acid metabolism
	Ferulate	Xenobiotics	Food component/plant
Serum only	Nicotinate ribonucleoside	Cofactors and vitamins	Nicotinate and nicotinamide metabolism
	Cholate	Lipid	Primary bile acid metabolism
	Beta-muricholate	Lipid	Primary bile acid metabolism
	Ferulic acid 4-sulfate	Xenobiotics	Food component/plant
	2-Oxindole-3-acetate	Xenobiotics	Food component/plant
	4-Methylcatechol sulfate	Xenobiotics	Benzoate metabolism
Fecal and serum	Phenylacetylglycine	Peptide	Acetylated peptides
	Glutarate (C5-DC)	Lipid	Fatty acid, dicarboxylate

### Microbiota-depended associative learning is associated with glial cell line-derived neurotrophic factor (GDNF) expression in the early postnatal brain

**Figure 8:**
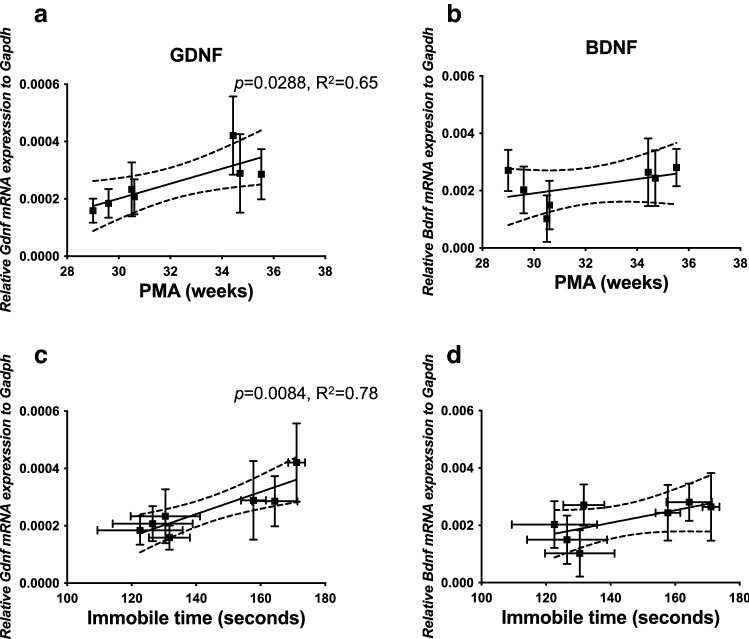
PMA-depended neurotrophic factor GDNF, but not BDNF, was associated with improved fear retention. Gene expression of GDNF levels examined by RT-PCR were significantly increased with increasing PMA (**a**). BDNF levels were not changed with PMA (**b**). Data are expressed as the ratio of the mRNA expression of the gene of interest relative to GAPDH mRNA expression. Data are presented as mean** ± **SEM and n = 5–13. Increased GDNF (**c**), but not BDNF (**d**), was significantly associated with increased immobile time in the second half of the cued testing in fear conditioning test.

To further investigate the molecular mechanisms by which microbiota regulate associative learning and memory in the fear conditioning test, we examined the cerebral gene expression of GDNF and brain-derived neurotrophic factor (BDNF) in pups at 2 weeks of age by RT-PCR. The 2 weeks old time point was chosen because both neurotrophic factors play key roles in early development, differentiation, synaptogenesis, and survival of neurons in the neonatal brain as well as neuronal plasticity and cognitive function. We demonstrate here that GDNF (Fig. 8a), but not BDNF (Fig. 8b), mRNA expression levels were associated with the PMA of the fecal samples. Furthermore, the increased GDNF levels were significantly correlated with the immobile time in the second half of the cued fear testing (Fig. 8c). BDNF levels were not associated with the immobile time (Fig. 8d).

## Discussion

From 23 to 40 weeks of gestation the human fetal brain gradually reaches critical developmental benchmarks^21^. After birth, the maturation process continues until 2–3 years of age when the brain reaches 90–95% of adult brain weight and synaptogenesis reaches its peak rate^22^. Preterm infants, especially those born at less than 32 weeks of gestational age and therefore deprived of the in utero developmental period, are at increased risk for adverse neurological outcomes with later cognitive and behavioral deficits^1,23–25^. In meta-analysis studies, poor neurodevelopment exhibited in motor skills, behavior, reading, mathematics and spelling were observed in preterm infants at primary school age^25^. When following up preterm infants to young adulthood (age 19), the impairment of cognitive function of preterm individuals persisted^26^. While many intrinsic and extrinsic factors determine the trajectory of brain development, studies have suggested that targeting the microbiome early in life might alter the early programming of the brain^9,27–29^. These observations revealed to us that preterm infants might be at specific risk for neurodevelopment deficits due to an initial immature microbiome associated with preterm birth. In this study, we specifically targeted the earliest colonization period by transfaunating pregnant GF mice with preterm infant fecal samples of different PMA and then studied the offspring’s adult behavior. To our knowledge the present study is the first to demonstrate that in GF mice transfaunated with a human preterm microbiome, and with early development of microbiota defined by PMA and with bacterial composition mimicking the preterm microbiota development, the microbiome primed a difference in associative learning in adult mice.

The strength of our model is to isolate the effect of microbiota at the earliest stage on neurodevelopment outcomes later in life. Our GF animals live in isolators in a gnotobiotic facility and are exposed to the same environment with one exception: colonization of different microbiota from human preterm infants at E16. The pups were reared naturally and stayed with the dams until weaning. The pups continued to live in the respective isolators in separated cages until the time points at which various experiments were assigned. We showed the fidelity of this colonization model by demonstrating that the microbiota of the pups at 2 and 4 weeks clusters together with the respective dams. Therefore, this model allowed us to investigate the direct impact of different microbiomes from preterm infants born at different GA and sampled at different PMA on adult behaviors.

PMA as the only significant and dominant factor in multifactorial analysis including delivery mode, feeding type, and antibiotic exposure has been previously shown to contribute significantly to the increase of species richness in preterm infants born between 24 and 30 weeks of GA^30^. Along this line of evidence, our current study demonstrated that colonizing mice at the earliest time point (around birth) with the fecal samples of human preterm infants at different PMA recapitulated the age-depended microbiota maturation phenotype in the mice. PMA of preterm donors was significantly positively correlated with the measurement of offspring fecal alpha-diversity including richness at 2 weeks of age.

We further identified several key PMA-depended taxonomic characteristics. First, PCA analysis demonstrated that Staphylococcus, *Streptococcus*, and *Stenotrophomonas* in < 31 weeks PMA groups contributed the separation from the > 31 weeks of PMA groups which featured *Enterobacter_D, Bifidobacterium, Lactococcus, Phocaeicola, Parabacteroides,* and *Escherichia*. These data agree with the current literature that the microbial community in preterm infants changes from dominance of *Staphylococcus* between PMA 25 and 30 weeks, *Enterococcus* from 30 to 35 weeks, and *Enterobacter* at 35 weeks. *Bifidobacterium* dominance starts to develop gradually only after 30 weeks PMA^5,20,31,32^. Secondly, there was an overall microbial taxonomic diversity (beta-diversity) difference among the different PMA groups in fecal samples from both 2 and 4 weeks of age. The observed differences in diversity were due to the increased abundance of *Clostridiaceae* and *Clostridium_P* in the later GA and PMA transfaunation groups in the 2 weeks fecal samples in contrast to the *Bacteroides, Phocaeicola, Bifidobacterium, Lactococcus, Enterobacter_D* in 4 weeks old fecal samples.

More importantly, we report here the first evidence that early maturation of microbiota, highlighted by bacterial diversity and dominance of the above mentioned key taxa led to improved performance in the cued fear conditioning test later in adult life. A previous study has shown that age (state of the maturation)-discriminatory taxa in healthy and stunted infants had direct impact on growth phenotypes^33^. We now show that increased PMA of fecal samples from preterm infants and PMA-depended diversity in early fecal samples of colonized mice projected better fear learning and memory in adult mice. We further demonstrate that not only the compositional differences, but also the abundance difference of early microbial taxa at the genus level, such as *Clostridioides* and *Clostridium_P* at 2 weeks of age and *Phocaeicola, Bifidobacterium, Lactococcus, Enterobacter_D* at 4 weeks of age, had long lasting effects in our study. The specific taxa identified in this study that were associated with fear retention could be the targets for future studies to investigate their contribution as single species or a consortia of species to improve neurological outcomes.

A few recent studies have suggested a role for the gut microbiota on fear retention and extinction using GF, antibiotic-treated specific pathogen free (SPF) and ex-GF in which a bacterial community was introduced to the GF mice at various ages^10,28,34^. GF mice exhibited reduced freezing during the cued memory retention test and the impaired function was related to a unique baseline transcriptome in the GF amygdala^34^. Our previous study demonstrated a reduced fear retention in GF mice when compared to SPF at 12 weeks of age^10^. Using several MRI methods, we documented that commensal microbiota regulate contextual memory and fear retention through white matter organization and myelination of several gray matter structures including the neocortex, hippocampus, brainstem and major white matter tracts including the corpus callosum, anterior commissure and internal capsule. One intriguing and perhaps more revealing study demonstrated that extinction learning deficits in fear conditioning tests were not reversible in GF mice after weaning when GF mice were colonized at either adult or weaning age^28^. However, when GF pups were colonized immediately after birth by fostering to SPF surrogate mothers, their fear extinction deficit was restored, indicating there is a critical developmental period before weaning during which microbiota can impact fear extinction learning and learning-related plasticity. With the previous studies comparing mice with or without commensal bacteria, our current study provides the first evidence that PMA-dependent early microbiome development has direct impact on adult fear behavior.

The age-dependent shift in the gut microbiota profile also shaped the metabolome in the offspring serum and fecal samples. Changes in a number of metabolites as the gut microbiota matured were associated with differences in fear learning and memory in our study. We identified 5-aminovalerate, phenol sulfate, 2,3-dihydroxy-2-methylbutyrate, ferulate, and ursodeoxycholate in fecal samples; nicotinate ribonucleoside, cholate, beta-muricholate, ferulic acid 4 sulfate, 2-oxindole-3-acetate, and 4-methylcatechol sulfate in serum samples; and phenylacetylglycine and glutarate (C5-DC) in both pools were associated with the altered fear retention.

Several of the metabolites we identified have been shown to be associated with brain function. Compared to SPF with normal fetal thalamocortical axonogenesis, 5-aminovalerate was significantly decreased in E14.5 fetal brains from embryos of GF and antibiotic-treated SPF dams^29^. In a study where fear extinction deficit was restored when GF pups were fostered to SPF surrogate mothers, phenyl sulfate levels in serum and fecal samples were also restored in GF pups^28^. Ferulate (ferulic acid) significantly repaired the spatial cognitive and memory deficits induced by ischemia in rats by reduced neuronal apoptosis and oxidative stress^35^. Recently, a large number of metabolites including the 5-aminovalerate, phenol sulfate and phenylacetylglycine identified in our study were profiled in postnatal brains^36^, suggesting that these microbial-derived metabolites might cross the blood–brain barrier to have direct effects on postnatal brain development. The role of most of these metabolites in regulating fear learning and memory are largely unknown. Future studies are warranted to investigate the age-associated shifts of microbiota metabolites on specific pathways related to associative learning and memory.

At the molecular level, both GDNF and BDNF play important roles in regulating learning and memory^37–40^. A reduction in endogenous GDNF levels in GDNF heterozygous mice has been associated with impaired spatial memory and learning^39^. Intracerebroventricular injection of GDNF attenuated anesthesia-surgery-induced fear learning and memory impairment in rats^40^. We observed that increased transcriptional levels of GDNF were associated with the PMA of the fecal microbiota and with better performance on the fear conditioning test, suggesting a potential early signaling interaction between microbiota maturation and GDNF in the brain. GDNF specifically promotes the development and survival of midbrain dopaminergic neurons^41,42^, motor neurons^43^, central noradrenergic neurons^44^, and cerebellar Purkinje neurons^45^ via its canonical GDNF family receptor α1(GFRα1)/RET receptor complex^46^, or an alternative neural adhesion molecule NCAM signaling pathways^47^. It would be of significant interest to investigate whether microbiota-mediated GDNF could specifically affect certain population of neurons and delineate the signaling pathways involved in future studies.

One limitation of our study is the common challenge in gut microbiome and brain development research of translating animal study results to humans. We isolated the mice in a gnotobiotic environment while the development of human infant microbiome will be shaped by many factors. However, our results demonstrated that the “degree” of early maturation of microbiome, simply signified by PMA, can predict behavioral outcome. This strengthens the notion that early microbiome development is critical for the neurodevelopment. However, the key taxa and metabolites identified that were related to improved associative learning ability will need to be tested directly in colonization or treatment experiments to establish causation. Transfaunating each experimental group with one human fecal sample and n ≥ 3 transfaunated animals in each PMA group are other limitations of this study. Although the number of donors is limited in this study, the study focus was directed at isolating the microbiome effect on long tern neurodevelopment based on their PMA and highlighting the importance of early intervention in the preterm population.

In conclusion, we have demonstrated that PMA-dependent maturation of the early gut microbiota affects associative learning and memory. Preterm infants born with gut microbiota immaturity might be at specific risk for impaired associative learning ability. The microbial characteristics including taxa and microbial metabolites identified in this study provide potential interventional targets to improve maturation of the microbiota in preterm infants and ultimately improve long term neurological outcomes.

## Methods

### Clinical study design

Patients were enrolled from The University of Chicago Comer Children’s Hospital, a level IV NICU in Chicago, Illinois, from 2015–2018. The study was approved by the Institutional Review Board. All methods were performed in accordance with the relevant guidelines and regulations. Informed consent for the study was obtained from infant parent(s). All infants enrolled were delivered between 27 and 34 + 1/7 weeks gestation and had no congenital anomalies (see Table 1). Patients with seizures were not included in the study shown at the last line in Table 1*.*

### Preparation of human fecal samples for transfaunation to GF mice

Transfaunation protocols were carried out as previously described^48^. Aliquots of frozen fecal samples from patients (100 mg per patient) in each PMA group prior to 2 weeks of life were resuspended under anaerobic conditions in 5 mL phosphate-buffered solution (PBS). Homogenates were clarified by 100 μM pore-diameter nylon filters (BD Falcon) before being stored in PBS containing glycerol (final concentration 15% v/v) at − 80 °C in 600 μL aliquots. To initiate microbial colonization, timed-pregnant GF eight to 9-week old mice (estimated between E15-17) were gavaged with 0.25 mL aliquots of fecal supernatant from each PMA group (n > 3 dams per group). Pups were delivered spontaneously and litters remained with the mother until weaning.

### Animals

All animal studies were approved by the Institutional Animal Care and Use Committee of the University of Chicago and Northshore University HealthSystem (NorthShore). GF C57/BL6J mice were maintained in the gnotobiotic facility of the Digestive Disease Research Core Center at the University of Chicago. All groups of mice were allowed ad-libitum access to NIH-31 GF chow and water. Mice were tested for behaviors at 12 weeks of age. Animals were transported to NorthShore in sterile containers 3–5 days before testing to allow an acclimation period. Upon arrival to NorthShore mice were housed in individually ventilated cages with HEPA filter until the behavioral testing began. Separate cohorts of pups were sacrificed at 2 weeks of age for the experiments listed below. All methods were performed in accordance with the relevant guidelines and regulations. The reporting in the manuscript follows the recommendations in the ARRIVE guidelines.

### Sample collection

Mice were euthanized under isoflurane followed by cardiac puncture for blood collection. Blood samples were allowed to clot for 15 min and spun down at 2000×*g* for 10 min. Supernatants were collected as sera and stored at − 80 °C for metabolomics analysis. Fecal samples of the pups were collected from the colons and stored at − 80 °C for sequencing analysis. Fecal samples from the dams were collected at the gnotobiotic facility. Brains of the pups were removed from the skulls and cerebrums were snap frozen on dry ice and stored at − 80 °C for molecular analysis.

### Microbial data analysis

Numbers of the fecal samples used in the following analysis were presented in Supplementary Table S5. Gr_1-7 corresponded to patient 1–7 in Table 1. Mouse fecal samples were submitted to the Microbiome Metagenomics Facility of the Duchossois Family Institute at the University of Chicago (Chicago, IL, USA) or the Environmental Sample Preparation and Sequencing Facility at the Argonne National Laboratory (Lemont, IL, USA) for genomic DNA extraction and subsequent 16S rRNA gene sequencing on the Illumina MiSeq platform. Sequence read depth was not significantly different between the two facilities (Welch’s *t* test *p* = 0.526; negligible Cohen’s D effect size of − 0.141) (see cluster analysis in Supplementary Fig. S8). A subset of the samples was sent to both facilities to ensure even study group distribution and control for differences in background contamination; microbial genera that were only present in one of the sequencing runs through comparison of these samples and were not present at a threshold of ≥ 0.1% composition in any of the other samples in the sequencing run were removed. This procedure was found to be satisfactory as equivalent samples within this subset clustered together irrespective of sequencing run. Sequencing data has been submitted to NCBI SRA: BioProject ID PRJNA767601.

Data processing was conducted using R statistical software version 3.6.2 and the R package DADA2 version 1.14.1 pipeline^49^. The GTDB database version 95 was used for sequence classification. After merging and decontaminating the sequencing runs as above, α-diversity metrics of richness and Shannon diversity were calculated using R package iNEXT version 2.0.20, and for β-diversity analysis, data were center-log ratio (CLR) transformed via R package ALDEx2 version 1.18.0 while taking the median of the Monte-Carlo instances as the value, which allows standard statistical testing to be applied to the inherently compositional data^50^. Principle component analysis (PCA) was completed using R package ropls version 1.18.8 at the genus level. Non-metric multidimensional scaling (NMDS) and the PERMANOVA statistical calculation were applied to the Aitchison distance matrix (Euclidean distance matrix of CLR transformed data) computed from the genus level data by R package vegan version 2.5.7. Statistically significant differences by gestational age and postmenstrual age between alpha diversity metrics and individual taxon abundances at the phylum, family and genus levels were determined from constructing linear mixed models with transfaunation group as a random effect by R package lme4 version 1.1.26 and *p* values derived from Satterthwaite’s method by R package lmerTest version 3.1.3. To correct for multiple testing, the Benjamini–Hochberg method was utilized to evaluate which tests passed a false-positive threshold of < 1%. The effect size reported was the marginal coefficient of determination for generalized mixed-effect models (variance explained by fixed effects) calculated from R package MuMIn version 1.43.17. All plots were generated by R package ggplot2 version 3.3.3.

### Behavioral studies

Behavioral testing was conducted as previously described^10^. Animal movements were recorded and processed with ANY-maze software (Stoelting Co., Wood Dale, IL).

#### Open field test

Animals were placed individually in the center of an open clear field box (61 × 61 cm), and their spontaneous motor activity recorded for the following parameters: mean speed, traveled distance, and time spent traveled in the center (40 × 40 cm) and border zones.

#### Elevated-plus maze

The elevated plus maze, made of white acrylic plastic, consisted of four arms (each 28 × 5 cm) and a central area (5 × 5 cm) elevated 50 cm above the floor. Two arms were open and two were closed with 15-cm-high walls made of the same material. Mice were individually placed in the center facing an open arm and allowed to explore for 5 min. The following behaviors were scored: time spent in the closed and open arms.

#### Social interaction test

Two social behaviors (social interaction and social memory/ novelty recognition) were quantified using a rectangular 3-chamber test that included a 20 × 45 × 30 cm middle chamber made of acrylic plastic, with two 10 × 10 cm openings leading to two separate (left and right) chambers of the same size, each containing a steel cage enclosure. Each mouse (experimental subject) was placed in the middle chamber and allowed to explore for 10 min, with the right chamber empty but an unfamiliar congener (Stranger I) (non-littermate control SPF mouse of the same gender, housed in a separate container) held in the steel cage enclosure in the left chamber. Social interaction was determined by measuring the time spent by the experimental subject around the cage holding the unfamiliar congener versus the right empty steel cage. To measure social memory (or novelty recognition), a new novel stimulus mouse (Stranger II) was subsequently placed in the previously empty right steel cage. The tested mouse was allowed to explore and interact for 10 min. The same parameters as above were measured to determine the preference of the experimental subject for Stranger I (Familiar) or Stranger II. The social chamber was wiped with 70% alcohol after each test.

#### Contextual and cued fear conditioning test

The contextual and cued fear conditioning tests the ability of mice to learn and remember an association between environmental cues and aversive experiences. In this test, mice were placed into a conditioning chamber and were given parings of a CS (an auditory cue) and an aversive US (an electric foot shock). The conditioning chamber consisted of opaque acrylic plastic 30 × 30 × 21 cm walls and a shocking grid on the floor. During the conditioning stage at day 1, mice were allowed to freely explore the chamber for 120 s. Thereafter, a white 55 dB noise auditory cue was presented as a CS for 30 s, and a 0.8 mA foot shock was given to the mice as an US continuously during the last 2 s of the sound. The presentation of CS-US was repeated three times per session (120, 240, and 360 s after the beginning of the conditioning). Following the final foot shock, the mice were left undisturbed in the chambers for 90 s. After the conditioning session had been completed, the mice were returned to the same conditioning chamber 24 h later and scored for freezing behavior to measure contextually conditioned fear (context test). The mice were placed in the conditioning chamber and were allowed to freely explore the chamber for 300 s without CS and US presentations.

The cued test was conducted on the same day 2 h after the context test. In this test, the shocking grid was removed and the walls of the chamber were covered with checkerboard pattern wallpaper, providing a novel context that was unrelated to the conditioning chamber. Mice were placed into the testing chamber for 3 min. At the end of the first 3 min, the CS auditory cue that had been presented at the time of conditioning was given to mice for 3 min. The fear conditioning chamber was wiped with 70% alcohol after each test. Fear memory was assessed based on freezing behavior to the conditioned cued or the contexts to which mice were previously exposed. The outcome variables were immobile time in the context test and during the first and last 30 s of the cued test.

### Metabolomics analysis

All samples were prepared and analyzed using an Ultrahigh Performance Liquid Chromatography–Tandem Mass Spectroscopy (UPLC–MS/MS) platform (Metabolon, Durham, NC). This platform utilized a Waters ACQUITY ultra-performance liquid chromatography (UPLC) and a Thermo Scientific Q-Exactive high resolution/accurate mass spectrometer interfaced with a heated electrospray ionization (HESI-II) source and Orbitrap mass analyzer operated at 35,000 mass resolution.

Raw data was extracted, peak-identified and QC processed using Metabolon’s hardware and software. Compounds were identified by comparison to library entries of purified standards or recurrent unknown entities. Metabolon maintains a library based on authenticated standards that contains the retention time/index (RI), mass to charge ratio (m/z), and chromatographic data (including MS/MS spectral data) on all molecules present in the library. Furthermore, biochemical identifications are based on three criteria: retention index within a narrow RI window of the proposed identification, accurate mass match to the library ± 10 ppm, and the MS/MS forward and reverse scores between the experimental data and authentic standards. The MS/MS scores are based on a comparison of the ions present in the experimental spectrum to the ions present in the library spectrum. Data were presented as scaled imputed log-transformed (ScaledIMP) ± S.E.M and used for statistical analysis.

### RNA isolation and real-time PCR

Total RNA from snap frozen brains were isolated using the RNeasy^®^ Plus Mini Kit (QIAGEN GmbH, Hilden, Germany). 500 ng of isolated total RNA was used to synthesize cDNA using RT^2^ First Strand Kit from QIAGEN. TaqMan probes and primers (Thermo Scientific) were used for gene of interests and the housekeeping gene *Gapdh*. Gene expression was normalized to the housekeeping gene and expressed as relative expression of experimental controls.

### Statistical analyses

Linear regression was used to generate fit curve in the offspring with values presented as the mean ± SEM. Benjamini–Hochberg procedure was used to correct for multiple comparisons in metabolite data. GraphPad Prism 6 software (GraphPad Software, Inc., La Jolla, CA, USA) was used and a *p* value ≤ 0.05 was considered significant.

### Ethics approval and consent to participate

Institutional Review Board approval (#16-1431) at the University of Chicago was obtained for the study and all participants were provided written informed consent. All animal procedures were approved by the Institutional Animal Care and Use Committees of the University of Chicago (No. 71703) and Northshore University HealthSystem (No. EH16-264) and performed strictly in accordance with approved Animal Care and Use Protocols (ACUPs) by the U.S. National Institutes of Health.

## Supplementary Information

Supplementary Information.

Supplementary Table S2.

Supplementary Table S3.

## Data Availability

Sequencing data was submitted at NCBI SRA with the BioProject ID: PRJNA767601. The data generated during the current study are available from the corresponding author on reasonable written request.

## Abbreviations

GA: Gestational age
PMA: Postmenstrual age
GF: Germ free
SPF: Specific pathogen free
GDNF: Glial cell line-derived neurotrophic factor
BDNF: Brain-derived neurotrophic factor
CS: Conditioned stimulus
US: Unconditioned stimulus
