# Supplementary material for: Early preterm infant microbiome impacts adult learning
Source: Sci Rep. 2022 Feb 28;12:3310. doi: 10.1038/s41598-022-07245-w (PMC8885646; doi:10.1038/s41598-022-07245-w)
Supplement: Supplementary file 1 — Supplementary Information. [file 41598_2022_7245_MOESM1_ESM.pdf]

Supplementary Information

Early preterm infant microbiome impacts adult learning

Jing Lu[1], Lei Lu[1], Yueyue Yu[1], Kaitlyn Oliphant[1], Alexander Drobyshevsky[2], Erika C. Claud[1]*

*Correspondence: eclaud@peds.bsd.uchicago.edu

Author details:

[1]Department of Pediatrics, University of Chicago, Pritzker School of Medicine/Biological Sciences Division, Chicago, Illinois, USA 60637

[2]Department of Pediatrics, NorthShore University HealthSystem Research Institute, Evanston, Illinois, USA 60202

Jing Lu: jlu7@peds.bsd.uchicago.edu

Lei Lu: llu@bsd.uchicago.edu

Yueyue Yu: yyu@bsd.uchicago.edu

Kaitlyn Oliphant: koliphant@bsd.uchicago.edu

Alexander Drobyshevsky: adrobyshevsky@northshore.org

Figure S1.

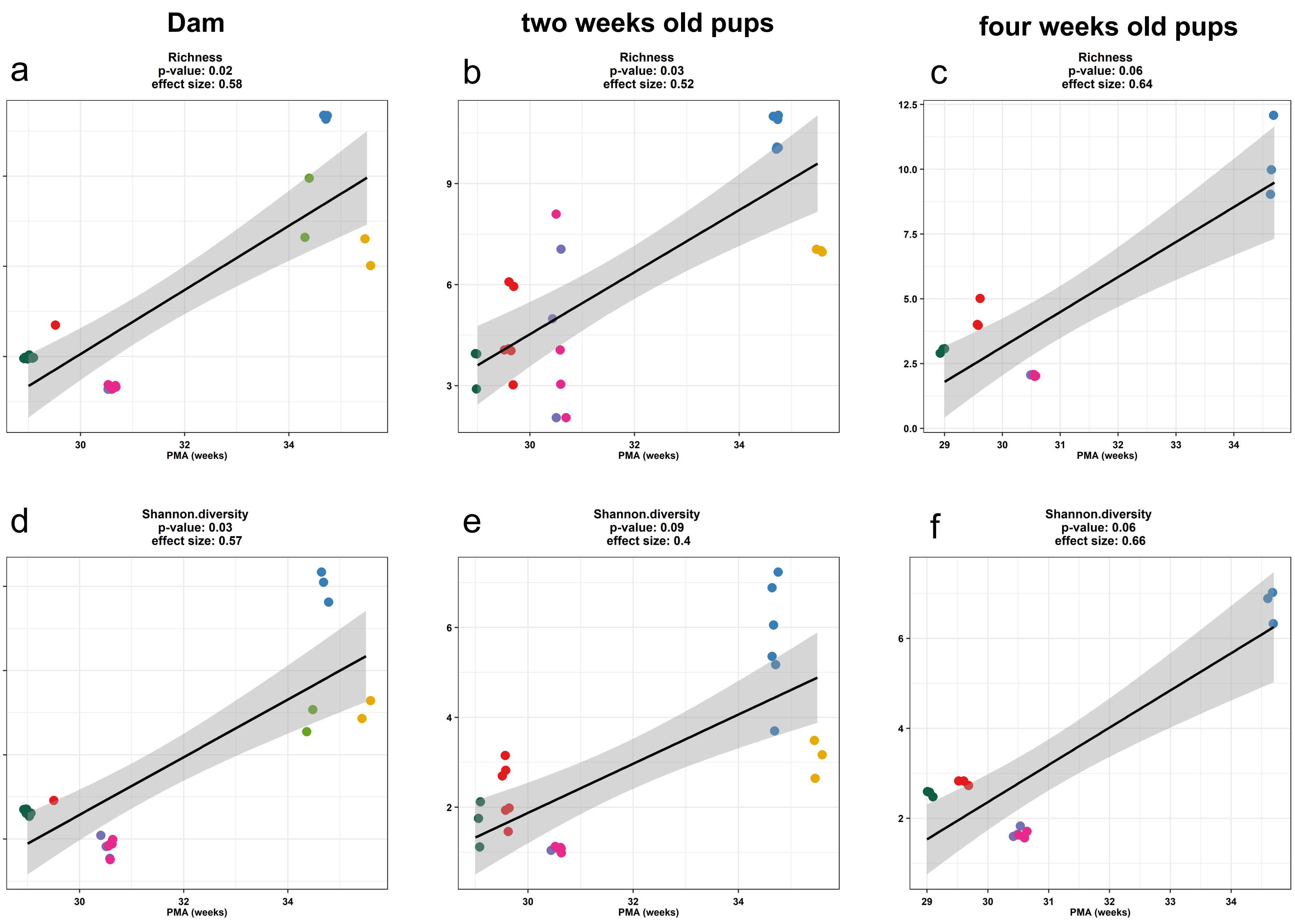

Dam
two weeks old pups
four weeks old pups
a
Richness
p-value: 0.02
effect size: 0.58
b
Richness
p-value: 0.03
effect size: 0.52
c
Richness
p-value: 0.06
effect size: 0.64
d
Shannon.diversity
p-value: 0.03
effect size: 0.57
e
Shannon.diversity
p-value: 0.09
effect size: 0.4
f
Shannon.diversity
p-value: 0.06
effect size: 0.66
PMA (weeks)
PMA (weeks)
PMA (weeks)
PMA (weeks)
PMA (weeks)
PMA (weeks)

Fig. S1. Increased PMA was positively correlated with fecal microbial alpha-diversity. Pearson correlation between PMA and richness in dam (a) (p=0.02), two weeks old pups (b) (p=0.03), and four weeks old pups (c) (p=0.06). Pearson correlation between PMA and Shannon diversity in dam (d) (p=0.03), 2 weeks old pups (e) (p=0.09), and 4 weeks old pups (f) (p=0.06). Shadow area indicates a 0.95 confidence interval.

Figure S2.

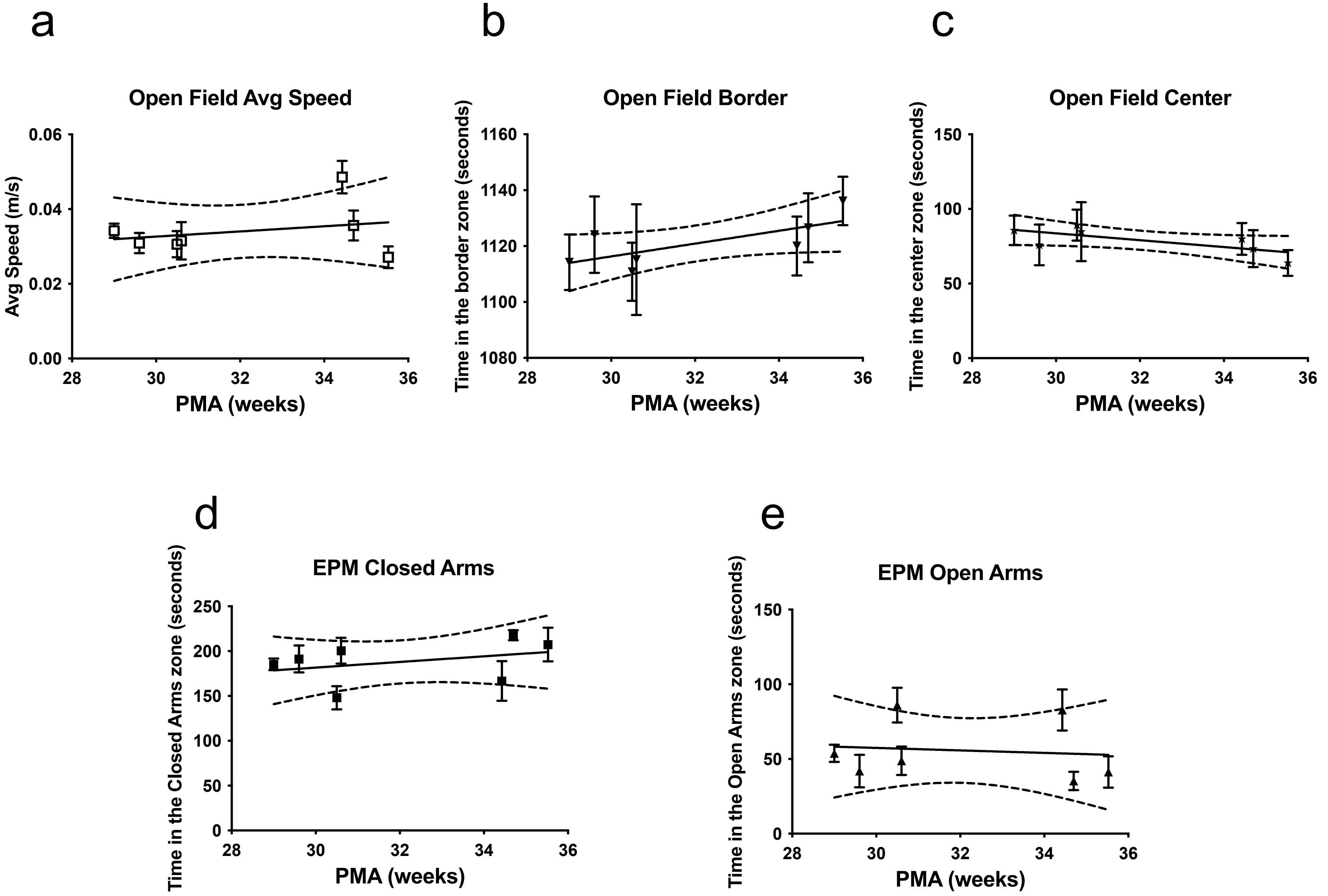
a
Open Field Avg Speed
Avg Speed (m/s)
0.06
0.04
0.02
0.00
28
30
32
34
36
PMA (weeks)
b
Open Field Border
Time in the border zone (seconds)
1160
1140
1120
1100
1080
28
30
32
34
36
PMA (weeks)
c
Open Field Center
Time in the center zone (seconds)
150
100
50
0
28
30
32
34
36
PMA (weeks)
d
EPM Closed Arms
Time in the Closed Arms zone (seconds)
250
200
150
100
50
0
28
30
32
34
36
PMA (weeks)
e
EPM Open Arms
Time in the Open Arms zone (seconds)
150
100
50
0
28
30
32
34
36
PMA (weeks)

Fig. S2. Locomotor and anxiety-like activities were not affected by PMA. Average speed animals travelled (m/s), time spent in the border region of the open field (seconds), time spent in the center quadrant of the open field (seconds), time spent in the open arms of the EPM (seconds), or time spent in the closed arms of the EPM (seconds) was not related to the PMA (a-e), respectively by Pearson's correlation coefficient analysis. Animal numbers used in each transfaunation groups were: gr_1=29, gr_2=8, gr_3=14, gr_4=6, gr_5=9, gr_6=11, and gr_7=7.

Figure S3.

a

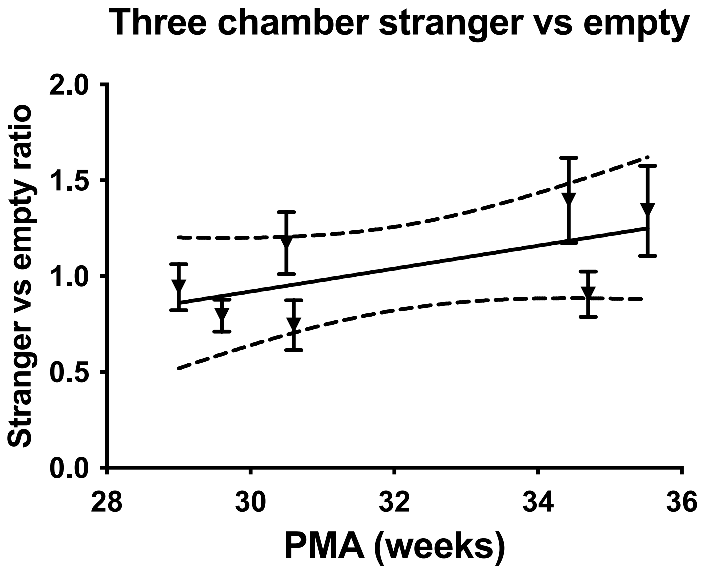
Three chamber stranger vs empty
Stranger vs empty ratio
2.0
1.5
1.0
0.5
0.0
28
30
32
34
36
PMA (weeks)

b

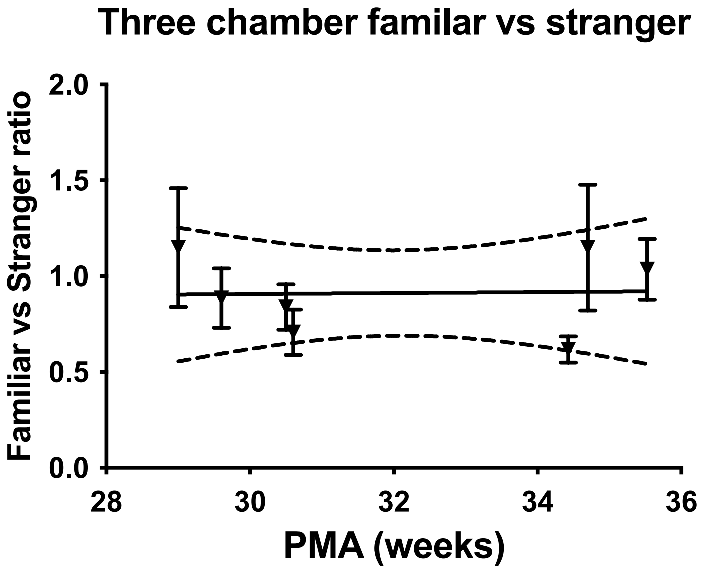
Three chamber familar vs stranger
Familiar vs Stranger ratio
2.0
1.5
1.0
0.5
0.0
28
30
32
34
36
PMA (weeks)

Fig. S3. Sociability and social novelty were not affected by PMA. In the sociability test, the ratio of the time a mouse’s head was in the cup zone in which a strange mouse is placed to that in an empty cup zone was not associated with changes of PMA (a) by Pearson's correlation coefficient analysis. In the test for social novelty, the ratio of the time a mouse’s head was in the cup zone in which a familiar mouse was placed to that in the cup zone of a stranger mouse was placed was not associated with changes of PMA (b) by Pearson's correlation coefficient analysis. Animal numbers used in each transfaunation groups were: gr_1=9, gr_2=8, gr_3=14, gr_4=6, gr_5=9, gr_6=10, and gr_7=7.

Figure S4.

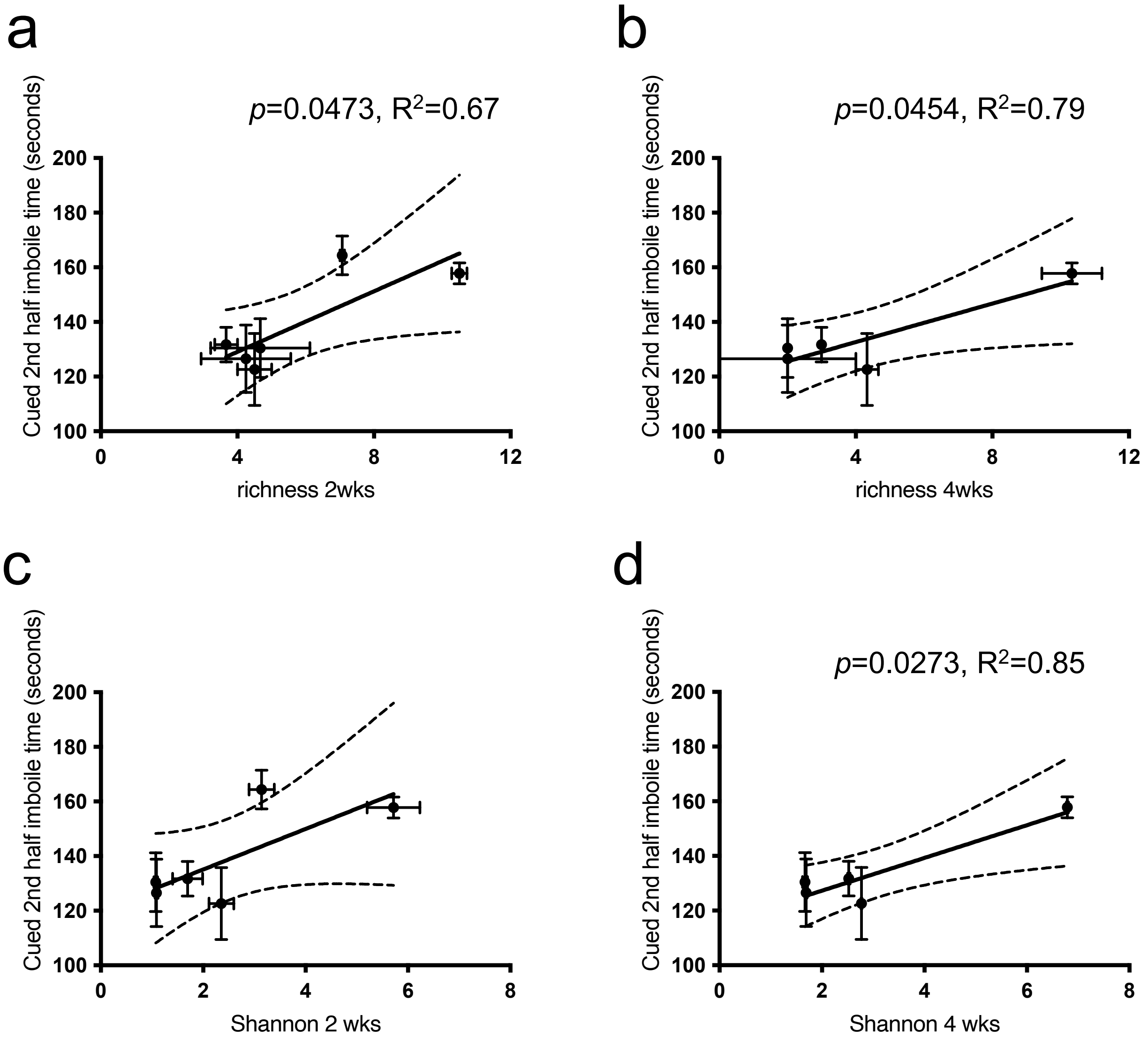
a
p=0.0473, R2=0.67
Cued 2nd half imboile time (seconds)
200
180
160
140
120
100
0
4
8
12
richness 2wks
b
p=0.0454, R2=0.79
Cued 2nd half imboile time (seconds)
richness 4wks
c
Cued 2nd half imboile time (seconds)
0
2
4
6
8
Shannon 2 wks
d
p=0.0273, R2=0.85
Cued 2nd half imboile time (seconds)
Shannon 4 wks

Fig. S4. Changes in richness and Shannon-diversity promoted cued fear retention in fear conditioning test. The richness of two (a) and four weeks (b) fecal samples showed a positive correlation with cued fear retention (immobile time in the second half of the cued fear conditioning test). Shannon-diversity of two weeks old fecal samples (c) was not associated with cued fear retention. Shannon-diversity of four weeks old samples (d) was correlated with cued fear retention. Pearson's correlation coefficient was considered significantly different at $p<0.05$.

Table S1. Fecal and serum samples subjected to metabolomic analysis. PMA (week) and number (n) of fecal and serum samples were listed.

	PMA (week)	Fecal samples (n)	Serum samples (n)
Early	29.6	5	6
	30.6	5	6
Late	34.7	5	5
	35.5	5	5

Table S2. List of fecal metabolites. Fecal samples were processed according to Metabolon's platform.

Table S3. List of serum metabolites. Serum samples were processed according to Metabolon's platform.

Table S4. Summary of Pearson correlation between PMA and metabolites. Pearson's correlation was considered significantly different at *p*<0.05. Benjamini–Hochberg procedure was used to correct for multiple comparisons.

**Fecal vs PMA**		**Fecal**	**Serum**
5-aminovalerate	P (two-tailed)	0.0012	0.0729
	R squared	0.9435	0.8595
phenol sulfate	P (two-tailed)	0.0012	0.1212
	R squared	0.9444	0.7723
2,3-dihydroxy-2-methylbutyrate	P (two-tailed)	0.0035	0.1108
	R squared	0.9514	0.7908
ferulate	P (two-tailed)	0.0019	0.0947
	R squared	0.9299	0.8195
ursodeoxycholate	P (two-tailed)	0.0015	0.0982
	R squared	0.9385	0.8133

**Serum vs PMA**		**Serum**	**Fecal**
nicotinate ribonucleoside	P (two-tailed)	0.0389	0.1044
	R squared	0.9237	0.5229
beta-muricholate	P (two-tailed)	0.0457	0.0982
	R squared	0.9106	0.8133

**Serum/Fecal vs PMA**		**Serum**	**Fecal**
glutarate (C5-DC)	P (two-tailed)	0.0290	0.0012
	R squared	0.9428	0.9434
cholate	P (two-tailed)	0.0361	0.0160
	R squared	0.9290	0.8008
phenylacetylglycine	P (two-tailed)	0.0460	0.0008
	R squared	0.9089	0.9554
ferulic acid 4 sulfate	P (two-tailed)	0.0187	0.0264
	R squared	0.9629	0.7471
2-oxindole-3-acetate	P (two-tailed)	0.0363	0.0037
	R squared	0.9288	0.9021

Figure 5S.

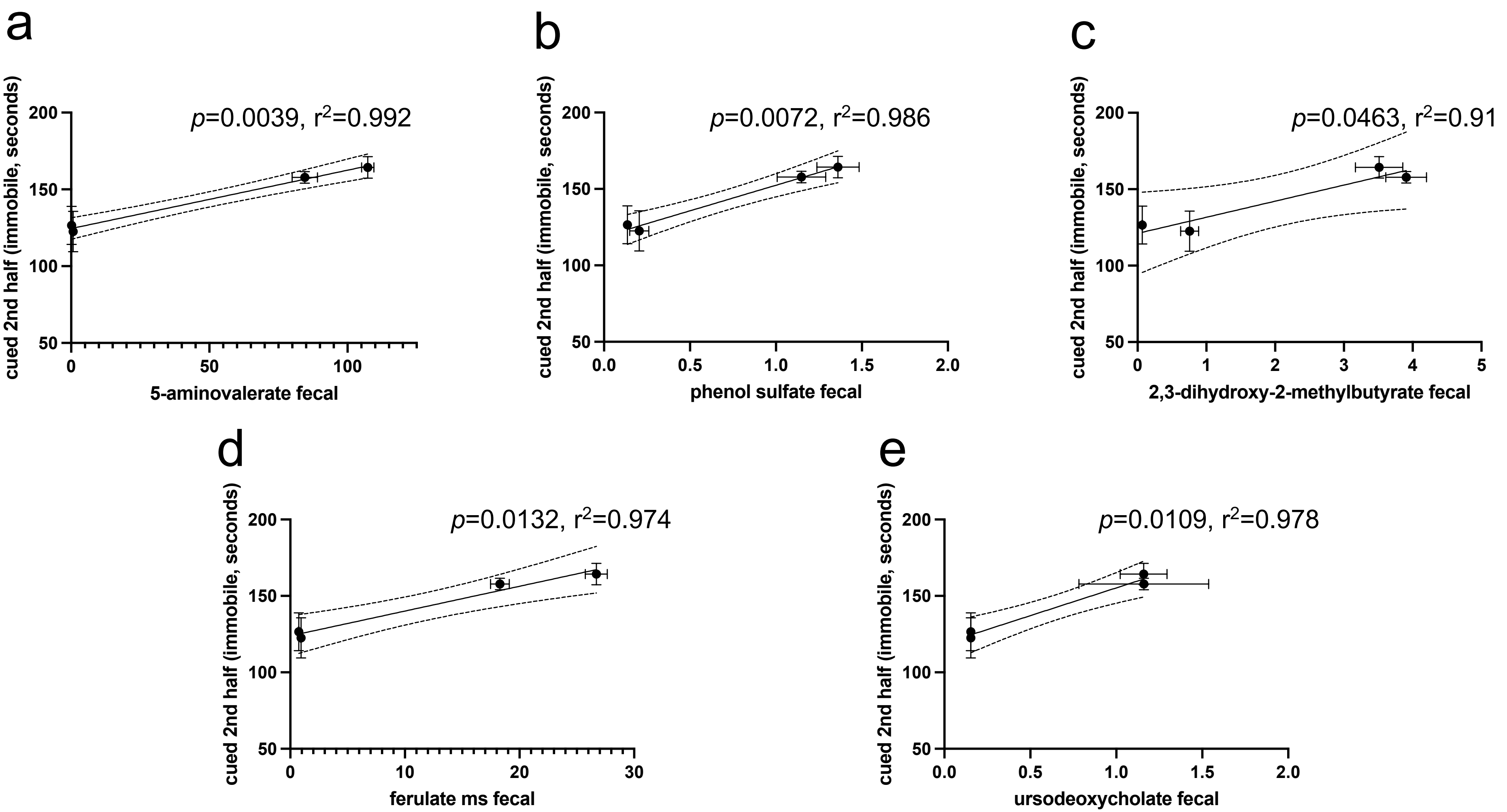
a
cued 2nd half (immobile, seconds)
p=0.0039, r2=0.992
200
150
100
50
0
50
100
5-aminovalerate fecal
b
cued 2nd half (immobile, seconds)
p=0.0072, r2=0.986
0.0
0.5
1.0
1.5
2.0
phenol sulfate fecal
c
cued 2nd half (immobile, seconds)
p=0.0463, r2=0.91
0
1
2
3
4
5
2,3-dihydroxy-2-methylbutyrate fecal
d
cued 2nd half (immobile, seconds)
p=0.0132, r2=0.974
0
10
20
30
ferulate ms fecal
e
cued 2nd half (immobile, seconds)
p=0.0109, r2=0.978
0.0
0.5
1.0
1.5
2.0
ursodeoxycholate fecal

Fig. S5. Fecal metabolic features contributed to the improved fear retention. Fecal 5-aminovalerate (a), phenol sulfate (b), 2,3-dihydroxy-2-methylbutyrate (c), ferulate (d), and ursodeoxycholate (e) were positively correlated with fear retention. ScaledIMP±S.E.M of metabolites was used to plot against the time in the second half of the cued fear conditioning test. All reached $p<0.05$ by Pearson's correlation analysis.

Figure 6S.

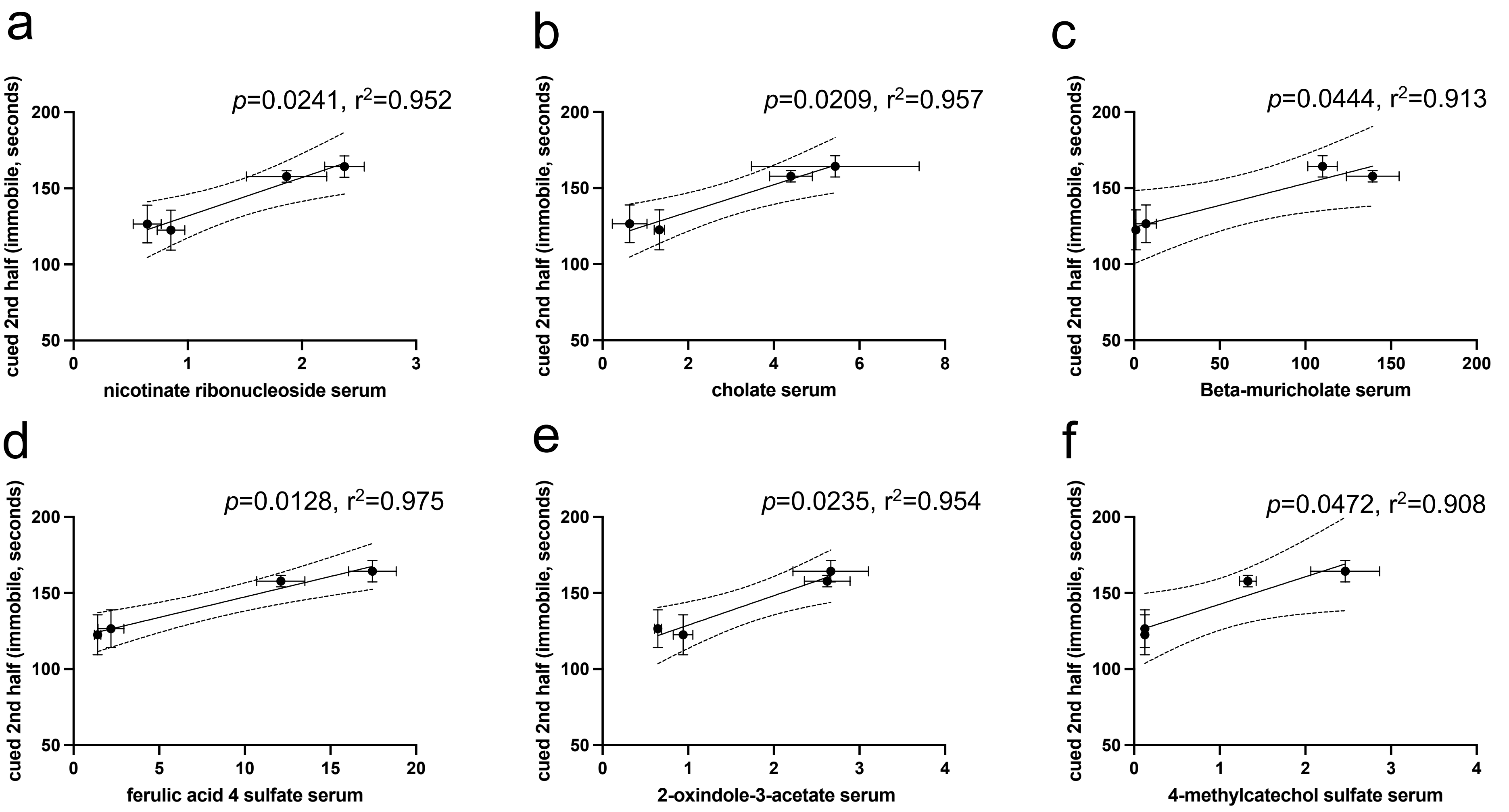
a
p=0.0241, r2=0.952
cued 2nd half (immobile, seconds)
nicotinate ribonucleoside serum
b
p=0.0209, r2=0.957
cued 2nd half (immobile, seconds)
cholate serum
c
p=0.0444, r2=0.913
cued 2nd half (immobile, seconds)
Beta-muricholate serum
d
p=0.0128, r2=0.975
cued 2nd half (immobile, seconds)
ferulic acid 4 sulfate serum
e
p=0.0235, r2=0.954
cued 2nd half (immobile, seconds)
2-oxindole-3-acetate serum
f
p=0.0472, r2=0.908
cued 2nd half (immobile, seconds)
4-methylcatechol sulfate serum

Fig. S6. Serum metabolic features contributed to the improved fear retention. Improved fear retention was positively significantly correlated with serum nicotinate ribonucleoside (a), cholate (b), beta-muricholate (c), ferulic acid 4 sulfate (d), 2-oxindole-3-acetate (e), and 4-methylcatechol sulfate (f). All reached $p<0.05$ by Pearson's correlation analysis.

Figure 7S.

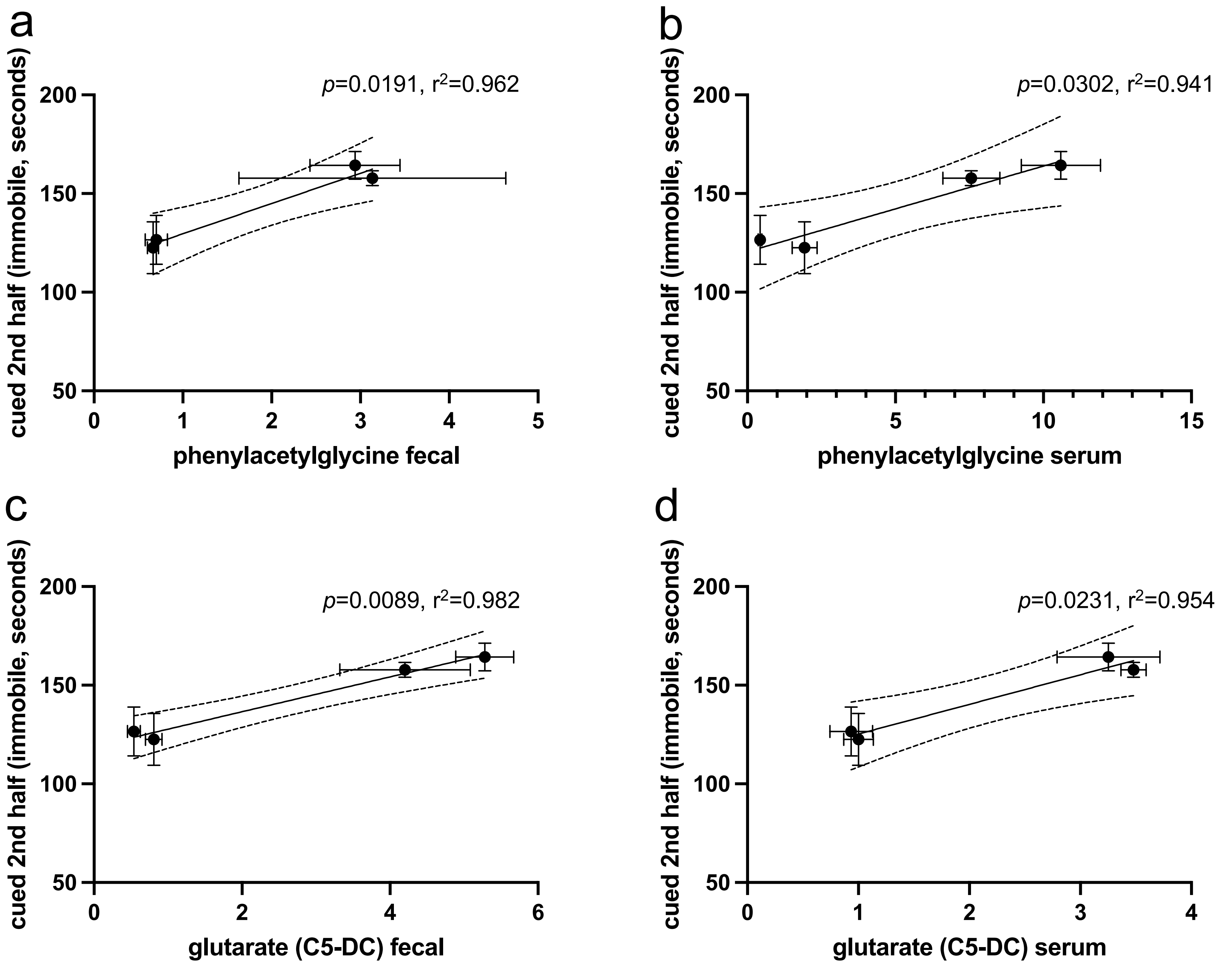

a
p=0.0191, r2=0.962
cued 2nd half (immobile, seconds)
200
150
100
50
0
1
2
3
4
5
phenylacetylglycine fecal
b
p=0.0302, r2=0.941
cued 2nd half (immobile, seconds)
200
150
100
50
0
5
10
15
phenylacetylglycine serum
c
p=0.0089, r2=0.982
cued 2nd half (immobile, seconds)
200
150
100
50
0
2
4
6
glutarate (C5-DC) fecal
d
p=0.0231, r2=0.954
cued 2nd half (immobile, seconds)
200
150
100
50
0
1
2
3
4
glutarate (C5-DC) serum

Fig. S7. Fecal and serum phenylacetylglycine (a and b) and glutarate (C5-DC) (c and d) levels were positively correlated with fear retention. All reached $p<0.05$ by Pearson's correlation analysis.

Table S5. Number of fecal samples sequenced in different transfaunation groups and time points. Number of fecal samples in each transfaunation group from dams (dam), two weeks old pups (2wpup), and four weeks old pups (4wpup) was presented.

Group	dam	2wpup	4wpup
gr_1	8	3	3
gr_2	1	6	3
gr_3	3	3	3
gr_4	5	4	3
gr_5	2	0	0
gr_6	3	6	3
gr_7	2	3	0

Figure 8S.

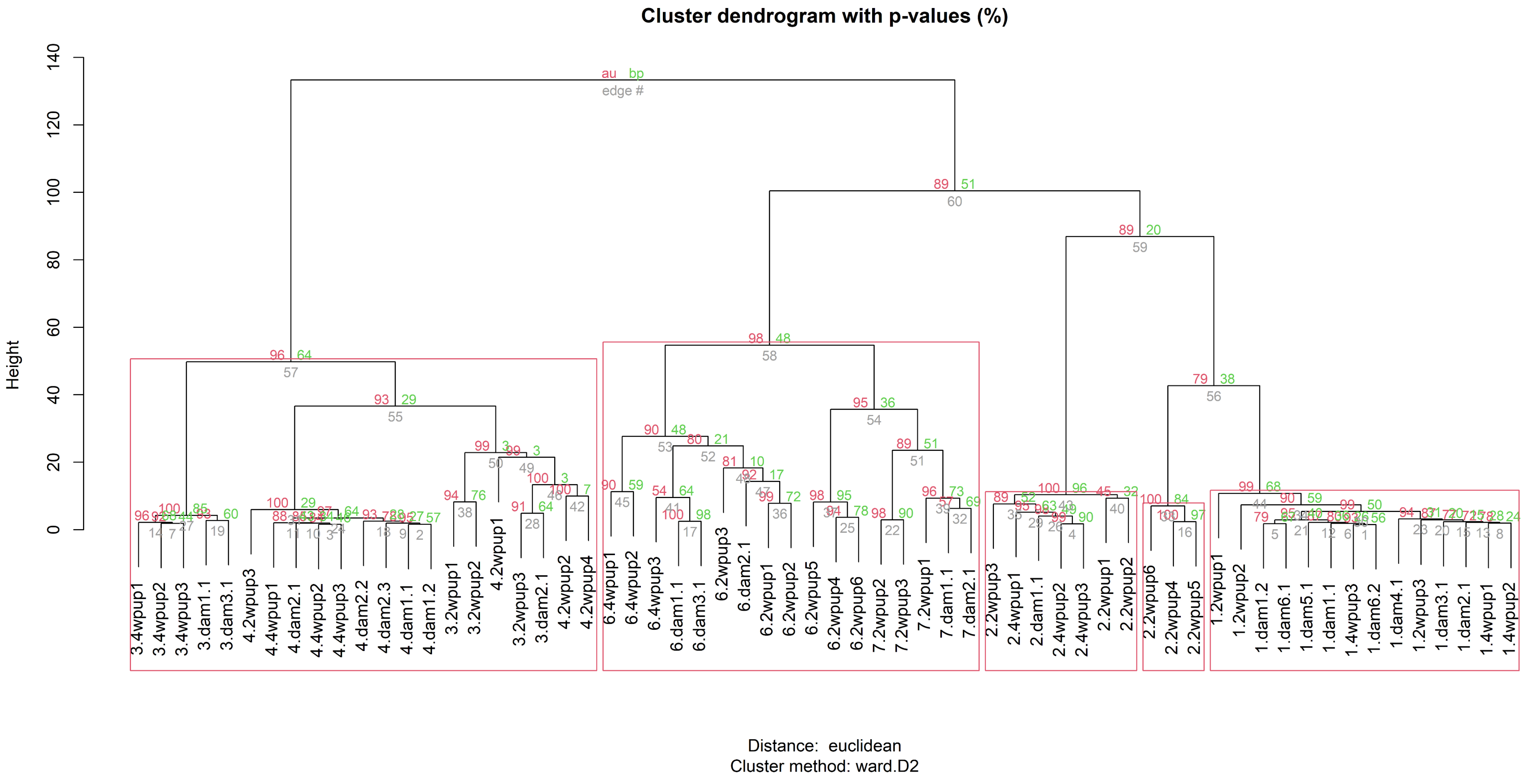

Cluster dendrogram with p-values (%)
Height
au bp
edge #
3.4wpup1
3.4wpup2
3.4wpup3
3.dam1.1
3.dam3.1
4.2wpup3
4.4wpup1
4.dam2.1
4.4wpup2
4.4wpup3
4.dam2.2
4.dam2.3
4.dam1.1
4.dam1.2
3.2wpup1
3.2wpup2
4.2wpup1
3.2wpup3
3.dam2.1
4.2wpup2
4.2wpup4
6.4wpup1
6.4wpup2
6.4wpup3
6.dam1.1
6.dam3.1
6.2wpup3
6.dam2.1
6.2wpup1
6.2wpup2
6.2wpup5
6.2wpup4
6.2wpup6
7.2wpup2
7.2wpup3
7.2wpup1
7.dam1.1
7.dam2.1
2.2wpup3
2.4wpup1
2.dam1.1
2.4wpup2
2.4wpup3
2.2wpup1
2.2wpup2
2.2wpup6
2.2wpup4
2.2wpup5
1.2wpup1
1.2wpup2
1.dam1.2
1.dam6.1
1.dam5.1
1.dam1.1
1.4wpup3
1.dam6.2
1.dam4.1
1.2wpup3
1.dam3.1
1.dam2.1
1.4wpup1
1.4wpup2
Distance: euclidean
Cluster method: ward.D2

Fig. S8. Cluster analysis of fecal samples. Hierarchical clustering analysis of the Aitchson distance matrix (Euclidean distance matrix of center-log ratio transformed data) generated from fecal microbial genera abundances with Ward's minimum variance linkage method. The statistically significant clusters (>=95%) are indicated by red rectangles, with p-values calculated from multiscale bootstrap resampling conducted by R package pvclust version 2.2-0. The approximately unbiased (AU) p-values for each edge are indicated in red text, with the ordinary bootstrap probability (BP) values in green text and cluster labels in grey text. Each sample is labeled with the convention "transfaunation group #"."sample type""sample number". Sample types are 2-week-old pup (2wpup), 4-week-old pup (4wpup) and dam. Dam sample numbers have the mouse number indicated first followed by the sample number (e.g., "7.dam2.1" is the first fecal sample from the second dam in transfaunation group 7), whereas each pup sample came from a unique pup. Pup sample #s 1-3 and all dam samples were 16S rRNA gene sequenced at the University of Chicago Duchossois Family Institute Microbiome Metagenomics Facility (Chicago, IL, USA), whereas pup sample #s 4-6 were 16S rRNA gene sequenced at the Argonne National Laboratory Environmental Sample Preparation and Sequencing Facility (Lemont, IL, USA).
